# Predictors for pregnancy outcomes in Romanian women with Type 1 Diabetes Mellitus: a prospective study

**DOI:** 10.1186/1758-5996-6-125

**Published:** 2014-11-22

**Authors:** Bogdan Timar, Romulus Timar, Alin Albai, Dana Stoian, Razvan Nitu, Marius Craina

**Affiliations:** Department III – Functional Sciences, “Victor Babes”, University of Medicine and Pharmacy, Timisoara, Romania; Department VII – Internal Medicine, “Victor Babes”, University of Medicine and Pharmacy, Timisoara, Romania; Department XII – Obstetrics and Gynecology, “Victor Babes”, University of Medicine and Pharmacy, Timisoara, Romania

**Keywords:** Pregnancy outcomes, Type 1 diabetes mellitus, Glycemic control

## Abstract

**Background:**

Type 1 diabetes mellitus in pregnant women is associated with an increased risk of congenital malformations, obstetric complications, neonatal morbidity, and mortality. Our aim was to evaluate which factors from the first trimester of pregnancy have a significant impact on the pregnancy outcomes of women with type 1 diabetes.

**Methods:**

We included 94 pregnant women with type 1 diabetes in this study. In these patients, we analyzed the influence of several diabetes-related parameters on the pregnancy outcome. We compared the parameters between two cohorts: those with successful pregnancies and those with adverse pregnancy outcomes, defined as spontaneous abortion or congenital malformations. The influence of several factors on the pregnancy outcome was assessed using multivariate and univariate logistic regressions.

**Results:**

The prevalence of adverse pregnancy outcomes was 28.7%, and was associated with poorer glycemic control (p <0.001), lower frequency of daily self-monitoring tests (p <0.001), smoking status (p <0.001), alcohol consumption (p <0.001), increased prevalence of chronic complications of diabetes, and the presence of ketosis. However, the adverse outcomes were not significantly associated with age, duration of diabetes, presence of thyroid disease, or body mass index. Furthermore, planned pregnancy was found to be a significant protective factor (odds ratio, 0.15; p <0.001).

**Conclusion:**

These results indicate that by carefully planning the pregnancy, ensuring optimal glycemic control, and eliminating habitual risk factors, the fetal risk in pregnancies among women with type 1 diabetes may decrease to a value similar to that noted in women without diabetes.

## Background

Type 1 diabetes mellitus (T1DM) in pregnant women is associated with a significant increase in the risk of congenital malformations, obstetric complications, neonatal morbidity, and mortality [1]. The frequency of major congenital malformations among fetuses of mothers with T1DM was estimated to be approximately 6–10%, which represents a 2–5-fold increase compared to the abnormalities noted in mothers without diabetes [2]. These increases in risk are primarily related to poor glycemic control during the first trimester of pregnancy, during which organogenesis takes place [3]; however, the likelihood hypoglycemic events and ketoacidosis should also not be overlooked in such cases [4].

Achieving optimal glycemic control without the occurrence of hypoglycemic events is one of the most important factors influencing pregnancy prognosis [5]. This is challenging because pregnancy itself results in an increase in the insulin resistance among women, even in women with T1DM—some reports have cited an increase in the daily insulin need of up to 40%. In addition, the daily insulin need was reported to fluctuate during the entire pregnancy period, primarily due to hormonal changes; previous studies have indicated the occurrence of at least two peaks in the insulin need, followed by decreases, during the pregnancy period [6]. Diet habits, particularly changes in the diet during pregnancy, should also be considered as factors that make good glycemic control difficult to achieve.

In 1989, the St. Vincent Declaration set a five-year aim to decrease the unfavorable outcomes in pregnant women with T1DM to the levels noted among pregnant women without diabetes [7]. However, although the fetal complications currently noted among pregnant women with T1DM have improved, the risk of severe and fatal outcomes is still significantly higher in pregnancies of women with T1DM [8].

Since until now there are no studies to analyze the pregnancy outcomes of the Romanian women with T1DM, in the present study, we aimed to analyze the pregnancies outcomes in this population and to evaluate which clinical and biological factors from the first trimester of pregnancy are significantly influencing the prognosis of these pregnancies.

## Methods

### Patients

We enrolled 94 pregnant women with T1DM between 2007 and 2012 in this observational study, using a population-based consecutive-case enrollment principle, at their first visit to either the Diabetes Clinic of the Emergency Hospital from Timisoara or Obstetrics Gynecology Clinic of the same hospital, if the visit was in the first trimester of pregnancy. The study design, protocol and informed consent form were reviewed and approved by the Ethics Committee of the Emergency Hospital Timisoara, Romania; all patients signed the informed consent form prior any study procedure or activity.

To assess the impact of the factors on the pregnancy outcomes, the patients were divided according to the pregnancy outcome into two cohorts: patients with adverse pregnancy outcomes (APO) (including those with fetal loss, spontaneous abortions, and congenital malformations) and those with normal births. The diagnosis of spontaneous abortion was established by the gynecologist which followed the pregnancy and the diagnosis of congenital malformations by the neonatologist respectively by the pathologist in case of major congenital malformations which led to perinatal death. We evaluated the differences in age, duration of diabetes, HbA1c level and body mass index (BMI), smoking status, alcohol consumption, presence of thyroid disorders, chronic complications of diabetes (retinopathy, neuropathy, and nephropathy), and the occurrence of ketosis in the first trimester of pregnancy or preeclamspia between the two groups.

During the pregnancy, 14 patients (14.9%) were treated with insulin pumps, whereas the other 80 patients received a basal-bolus insulin regimen, involving two injections of neutral protamine Hagedorn and three injections with regular insulin. All patients received diet counseling from a specialized dietician during the pregnancy. With regard to self-monitoring of blood glucose values, although the guidelines recommend the need for extensive glucose profile examinations, the Romanian Health Insurance company only covers the cost of one glucose strip daily; the patients need to bear the cost of any additional strips used.

### Clinical, anthropometric, and laboratory data

Data regarding patient age, history of prior pregnancy loss, and smoking status were collected from the patients’ medical records. The duration of diabetes was defined as the time from the date of the first insulin injection until the diagnosis of the pregnancy (confirmed by gynecological examination). The HbA1c level was measured using a NGSP-standardized and DCCT-compliant immune-turbidimetric assay (Roche), having an inter-measurement coefficient of variation of 1.64% according to manufacturer’s specifications. The screening for ketosis was performed using Siemens Multistix 10 SG urine dipsticks. In case of a positive dipstick test for urine ketones total serum levels of ketone bodies were measured, a value higher than 1 mmol/L being considered a positive diagnosis for ketosis. Urine ketones measurement was performed as a screening method at every visit and also every patient was provided with dipsticks for home measurement. In case of a positive test for urine ketosis at home, patients were instructed to contact their diabetologist and to come to hospital for serum ketones measurement and treatment readjustment. Screening for thyroid diseases was performed in all the patients by measuring the TSH and FT4 levels, and via a thyroid ultrasonography examination. Diabetic retinopathy was diagnosed based on the results of ophthalmological examination and eye fundus examination. The Michigan Neuropathy Screening Instrument (MNSI) was used to diagnose diabetic neuropathy; an MNSI score of >2.5 was representative of a positive diagnosis [9]. Moreover, for the diagnosis of diabetic nephropathy, spot urine albumin/creatinine ratio tests were performed, and 3 mg/mmol was considered as the threshold for clinically significant albuminuria. Alcohol consumption was assessed by using a questionnaire adapted from the AUDIT-C screening tool proposed by the ACQUIP study group; a score of ≥8 was considered as the threshold for alcohol abuse [10]. Planned pregnancy was defined in cases where the women clearly intended to become pregnant, made extensive lifestyle preparations, and made at least one visit to a gynecologist and diabetologist prior to conception.

### Statistical analysis

Data were collected and analyzed using the SPSS v.17 software suite (SPSS Inc. Chicago, IL, USA). Data are presented as mean ± standard deviations for continuous variables with Gaussian distribution, median (interquartile range) for continuous variables without Gaussian distribution, or percentages for categorical variables. The lower and upper limits of the 95% confidence intervals (CI), used to estimate the prevalence, were calculated according to Wilson’s procedure for variables with Poisson distribution. Moreover, the 95% CI for odds ratio (OR) was calculated according to the mid-p method for binomial distributions [11].

To assess the significance of the differences between groups, the Student *t*-test (means, Gaussian populations), Mann-Whitney test (medians, non-Gaussian populations), and Fisher’s exact test (proportions) were used. Continuous variable distributions were tested for normality using D’Agostino’s and Pearson’s test, and for equality of variances using Levene’s test. For evaluating the involvement of one or more confounding factors in dichotomous outcomes, univariate and multivariate logistic regression models were established; the goodness of fit was estimated using Nagelkerke’s R^2^ method.

A p value of <0.05 was considered as the threshold for statistical significance.

## Results

The patients’ baseline characteristics are presented in Table 1.

**Table 1:** **Patient**’**s baseline characteristics**

Age*	26 [6]
Diabetes duration*	8 [10]
HbA1c (%) in the 1^st^ trimester of pregnancy^†^	7.8 ± 1.4
BMI (kg/m^2^) in the 1^st^ trimester of pregnancy^†^	23.5 ± 3.4
Daily insulin dose (U/kg)^†^	0.85 ± 0.25
Daily self-monitoring blood glucose tests^†^	2 [2]
Insulin-pump treatment^‡^	14.9% (14)
Smokers^‡^	44.7% (42)
Alcohol abuse^‡^	8.5% (8)
Thyroid disease^‡^	11.7% (11)
Diabetic retinopathy^‡^	23.4% (22)
Diabetic neuropathy^‡^	11.7% (11)
Diabetic nephropathy^‡^	4.3% (4)
Planned pregnancy^‡^	64.9% (61)
Ketosis in the 1^st^ trimester of pregnancy^‡^	21.3% (20)
Preeclampsia events (%)	16.0% (15)

*Distributions are not Gaussian. Data is presented as median and [interquartile range].

^†^Data are presented as mean ± standard deviation.

^‡^Data are presented as percentages.

In total, 27 cases of APO were observed, leading to a prevalence of 28.7%; of these cases, 21 (77.8%) were caused by spontaneous abortions and 6 (22.2%) were caused by congenital malformations: 4 cardiovascular malformations, one hydrocephaly and one esophageal atresia. No maternal deaths or major maternal complications were recorded. APO was significantly associated with poor glycemic control in the first trimester of pregnancy (average HbA1c higher by 1.69 percentage points; p <0.001), fewer daily blood glucose self-monitoring tests (median, 1 vs. 3 tests; p <0.001), smoking status (p <0.001), alcohol consumption (p <0.001), increased prevalence of chronic complications of diabetes (retinopathy, p = 0.016; neuropathy, p = 0.012; nephropathy, p = 0.037), and the presence of ketosis during the first trimester of pregnancy (p <0.001). However, APO was not significantly associated with age, duration of diabetes, BMI, preeclampsia events, and the presence of thyroid disorders (Table 2). Planned pregnancy was found to be a significant protective factor against the risk of APO (OR = 0.15; 95% CI, 0.05– 0.38; p <0.001). In the study group, no severe hypoglycemic events were recorded.

**Table 2:** **Comparison between groups**: **pregnancies with adverse outcomes vs. pregnancies without adverse outcomes**

	Without APO	With APO	p
Number of cases	67 (71.3%)	27 (28.7%)	-
Age*	26 [5]	25 [9]	0.212
Diabetes duration*	8 [11]	9 [7]	0.259
HbA1c (%) in the 1^st^ trimester of pregnancy^†^	7.33 ± 0.92	9.02 ± 1.6	<0.001
BMI (kg/m^2^) in the 1^st^ trimester of pregnancy^†^	23.32 ± 2.88	23.82 ± 4.35	0.586
Daily self-monitoring blood glucose tests*	3 [3]	1 [2]	<0.001
Smokers^‡^	32.8% (22)	74.1% (20)	<0.001
Alcohol abuse^‡^	0% (0)	29.6% (8)	<0.001
Thyroid disease^‡^	13.4% (9)	7.4% (2)	0.503
Diabetic retinopathy^‡^	16.4% (11)	40.7% (11)	0.016
Diabetic neuropathy^‡^	6.0% (4)	25.9% (7)	0.012
Diabetic nephropathy^‡^	1.5% (1)	7.4% (2)	0.037
Planned pregnancy^‡^	79.1% (53)	29.6% (8)	<0.001
Ketosis in the 1^st^ trimester of pregnancy^‡^	10.4% (7)	48.1% (13)	<0.001
Preeclampsia events^‡^	14.9% (10)	18.5% (5)	0.757

*Distributions are not Gaussian. Data is presented as median and [interquartile range]; p was calculated with Mann-Whitney *U* test.

^†^Data is presented as mean ± standard deviation; p was calculated with t-student test.

^‡^Data is presented as percentages; p was calculated with chi-square test.

Continuous variables distributions were tested for normality using Shapiro-Wilk test and for equality of variances with Levene’s test.

*APO* – Adverse pregnancy outcome.

*HbA1c* - Hemoglobin A1c.

*BMI* – Body Mass Index.

In the univariate logistic regression model, we analyzed the impact of glycemic control during the first trimester of pregnancy--using the HbA1c value--on the prognosis of pregnancy. We noted an increase in the risk of APO (OR = 3.07; 95% CI, 1.83–5.15; p <0.001) for every 1 percentage point increase in the HbA1c value.

**Figure 1:**
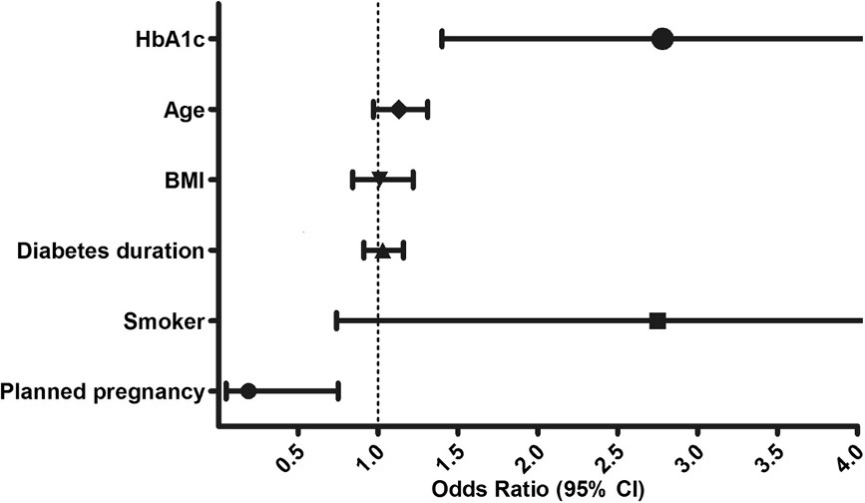
**Multivariate risk analysis for APO**; **predictors from the first trimester of pregnancy.** HbA1c – Hemoglobin A1c (risk is expressed per one percentage point increase); Age (risk is expressed per one year increase); BMI – Body Mass Index (risk is expressed per one kg/m^2^ increase); Diabetes duration (risk is expressed per one year increase); Smoker (risk is expressed as dichotomous variable); Planned pregnancy (risk is expressed as dichotomous variable).

The multivariate logistic regression model indicated that increases in the HbA1c level during the first trimester of pregnancy (OR = 2.78; p = 0.03) and planning of the pregnancy (OR = 0.19; p = 0.02) were significant factors that independently influenced APO (Table 3, Figure 1).

**Table 3:** **Predictors for APO in women with T1DM** (**multivariate logistic regression model**; **Nagelkerke R** ^**2**^ = **0.565**)

Predictor	OR	95% CI	p
HbA1c (*per percentage point*)*	2.78	1.4 to 5.45	0.03
Age (*per one year*)	1.13	0.97 to 1.31	0.11
BMI (*per kg*/*m* ^*2*^)	1.01	0.84 to 1.22	0.91
Diabetes duration (*per one year*)	1.03	0.91 to 1.16	0.64
Smoking status (*dichotomous*)	2.74	0.74 to 10.17	0.13
Planned pregnancy (*dichotomous*)*	0.19	0.048 to 0.75	0.018

*Predictor is significant both independently and as a co-factor.

## Discussion

To our knowledge, the present study is the first to analyze the influence of different potential risk factors for severe or fatal pregnancy outcomes in Romanian women with T1DM. Although a series of related risk factors that have already been reported in the literature may be applicable to the Romanian population, the risk factors may be different in Romanian women with T1DM due to social and economic characteristics among this group since the World Health Organization reports a prevalence of congenital malformations in general population of Romania as being 22 per 1,000 pregnancies, compared to an average of 3.19 per 1,000 in the European Region [12]. Another strength of our study is that we enrolled all the pregnancies of women with T1DM from our region, during a six year timeframe. The most important limit we identified is that the results are reflecting only the situation from the West part of Romania, being possible that some differences exists in other regions.

In the present study, involving a representative group of pregnant Romanian women with T1DM, we noted that the findings regarding pregnancy prognosis were similar to those obtained in other countries such as England, Northern Ireland, or Wales [13], but were worse compared to those found in Denmark, Italy or the Netherlands [14–16]. An important observation is that the value representing APO, among these studies, was strongly correlated with the presence or absence of the factors already identified in the present study as significant factors for pregnancy outcome. Also, when we compared our results with results found in similar studies [13–16], excepting ones from the England, Northern Ireland and Wales, we observed the existence of a strong correlation between the prevalence of APO in pregnancies of women with T1DM on one hand and the prevalence of APO in general population on the other hand, the prevalence of APO in pregnancies of women with T1DM being almost proportional with the prevalence of APO in general population of these countries [12].

The early planning of pregnancy is one such factor, which is one of the most important factors in the present study and is also mentioned in other studies [17, 18]. Pregnancy planning is an important component not only in women with T1DM, but also in the general population; however, its role is augmented, due to a series of specific characteristics in the former group [19]. In this group of women with T1DM, to decrease both the maternal and fetal risks, a multidisciplinary approach (particularly involving gynecology and diabetology specialists) is required, along with the consideration of certain factors, of which the important ones include achieving an optimal glycemic control before pregnancy, psychological support, and awareness of the features of the metabolic state during pregnancy; we are already aware that achieving glycemic control in pregnant women is more challenging due to the hormonal and dietary habit changes during this period [19, 20]. All these changes lead to a variable increase in the insulin need during the pregnancy, thus requiring stricter self-monitoring of blood glucose and concomitant adjustments of insulin doses [6, 21]. With regard to pregnancy planning, an important measure adopted--not only among those with T1DM--is the cessation of alcohol consumption and smoking prior to the pregnancy. In the present study, both smoking status and alcohol consumption were associated with an augmented fetal risk. Furthermore, through multivariate logistic regression analysis, lack of pregnancy planning was found to be a cofactor for fetal risk, along with the abovementioned factors, indicating that timely planning has an indirect role (the results being derived from planning interventions) and is also a significant independent factor protecting against fetal risk.

The importance of the factors identified is underlined by their independent nature of influence. In the present study, achieving good glycemic control was found to be a protective factor against adverse pregnancy outcomes; however this decrease in the risk is probably strongly related to an improved glycemic control obtained in the patients which self-monitored their blood glucose in a more appropriate way. We noted that increased levels of HbA1c during the first trimester of pregnancy, during which organogenesis occurs, are associated with significant increases in the fetal risk. Considering that the insulin need fluctuates during this period, the glucose levels require frequent measuring for adjusting the insulin doses. Thus, adequate self-monitoring during the entire pregnancy period is essential to achieve good glycemic control.

The impact of habitual risk factors such as smoking status and alcohol consumption has been already identified for pregnancies that are not complicated by diabetes [22, 23]; the present results suggest that these factors have a greater influence in pregnant cases with T1DM. The increased proportion of smokers and women who consumed alcohol during the pregnancy may contribute to the higher prevalence of negative outcomes, as compared to other studies wherein the fetal loss rate, along with the incidence of these two factors, was lower. Thus, the cessation of alcohol consumption and smoking at a considerable duration prior to pregnancy appears to be mandatory in women with T1DM. In our study the BMI proved to have no significant impact on the pregnancy outcome, the results being in contrast with ones found by Persson et al. which described a worsening of the pregnancy prognosis in women which had an increased BMI prior pregnancy in a cohort of women with T1DM [24]. Probably, our results revealed no significant impact because the proportion of obese and overweight patients was significantly smaller compared to Persson’s study, thus in our study the occurrence of a type 2 statistical error being possible, our cohort containing only 4 obese patients prior pregnancy.

Another significant risk factor in the present study is the presence of chronic (retinopathy, nephropathy, and neuropathy) and acute (ketosis) complications of diabetes. In the group of patients with negative outcomes, we observed significant increases in the proportion of patients with complications. However, after excluding these factors from our logistic regression model, we noted that the duration of diabetes and the mother’s age did not have a significant impact on the pregnancy outcome. Nevertheless, the duration of diabetes is known to be correlated with the occurrence of complications, and may thus be indirectly related to pregnancy prognosis [25].

## Conclusions

Based on the current findings, the presence of T1DM may be considered as only a fetal and not a maternal risk factor. In addition, we did not observe any major maternal complications or deaths in our cohort.

After analyzing the results of the present study and evaluating them in the context of other published findings, we conclude that by achieving good glycemic control and thus avoiding the occurrence of hypoglycemic or ketosis events, as well as by eliminating the habitual risk factors, the aim of the St. Vincent declaration of 1989 may be realized. Moreover, the results suggest that, by implementing these measures, the fetal risk in women with T1DM can be decreased to a value that is similar to that in women without diabetes.

## Abbreviations

T1DM: Type 1 Diabetes Mellitus
APO: Adverse pregnancy outcomes
BMI: Body mass index
HbA1c: Hemoglobin A1c
MNSI: Michigan neuropathy score instrument
CI: Confidence interval.
